# Thallium hyperaccumulation status of the violets of the Allchar arsenic–thallium deposit (North Macedonia) confirmed through synchrotron µXRF imaging

**DOI:** 10.1093/mtomcs/mfad063

**Published:** 2023-10-17

**Authors:** Ksenija Jakovljević, Tomica Mišljenović, Katerina Bačeva Andonovska, Guillaume Echevarria, Alan J M Baker, Dennis Brueckner, Antony van der Ent

**Affiliations:** Institute of Botany and Botanical Garden, Faculty of Biology, University of Belgrade, Belgrade, Serbia; Institute of Botany and Botanical Garden, Faculty of Biology, University of Belgrade, Belgrade, Serbia; Research Center for Environment and Materials, Macedonian Academy of Sciences and Arts, Skopje, North Macedonia; Université de Lorraine, INRAE, LSE, F-54000, Nancy, France; Centre for Mined Land Rehabilitation, Sustainable Minerals Institute, The University of Queensland, Brisbane, Queensland, Australia; Econick, Nancy, France; Université de Lorraine, INRAE, LSE, F-54000, Nancy, France; Centre for Mined Land Rehabilitation, Sustainable Minerals Institute, The University of Queensland, Brisbane, Queensland, Australia; Econick, Nancy, France; Deutsches Elektronen-Synchrotron DESY, Hamburg, Germany; Université de Lorraine, INRAE, LSE, F-54000, Nancy, France; Centre for Mined Land Rehabilitation, Sustainable Minerals Institute, The University of Queensland, Brisbane, Queensland, Australia; Econick, Nancy, France; Laboratory of Genetics, Wageningen University and Research, Wageningen, The Netherlands

**Keywords:** arsenic, excluder, hyperaccumulator, metallophyte, synchrotron-based X-ray fluorescence microscopy (XFM), thallium

## Abstract

The abandoned Allchar Mine in the Republic of North Macedonia is a globally unique deposit with the highest known grades of thallium (Tl) and arsenic (As) mineralization. We aimed to determine the distribution of As and Tl in whole dehydrated shoots of the three *Viola* taxa using synchrotron micro-X-ray fluorescence analysis. Additionally, soil and plant organ samples were collected from all three *Viola* taxa at the Allchar site and analysed using inductively coupled plasma–atomic emission spectrometry. Concentrations of Tl were extremely high in all three *Viola* taxa (up to 58 900 mg kg^−1^), but concentrations of As were highly variable with *V. tricolor* subsp. *macedonica* and *V. allchariensis* having low As (up to 20.2 and 26.3 mg kg^−1^, respectively) and *V. arsenica* having the highest concentrations (up to 381 mg kg^−1^). The extremely high Tl in all three species is endogenous and not a result of contamination. Arsenic in *V. tricolor* subsp*. macedonica* and *V. allcharensis* is strongly affected by contamination, but not in *V. arsenica* where it appears to be endogenous. The pattern of As enrichment in *V. arsenica* is very unusual and coincides with Ca-oxalate deposits and Br hotspots. The results of this study could form the basis for more detailed investigations under controlled conditions, including plant dosing experiments.

## Graphical Abstract

**Graphical Abstract:**
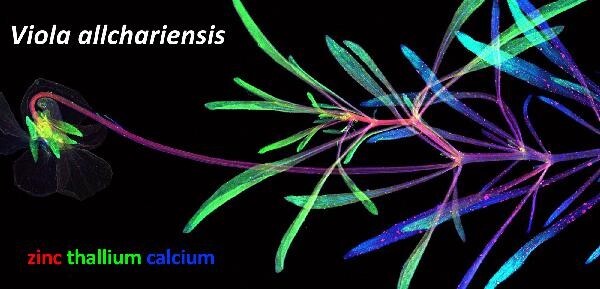
*Viola allchariensis* is an endemic species from Allchar in North Macedonia that has been found to hyperaccumulate Tl. This study also revealed Tl hyperaccumulation in two other *Viola* taxa from the same area, whose endogenous origin was confirmed by synchrotron micro-X-ray fluorescence analysis.

## Introduction

Thallium (Tl) and arsenic (As) are the most toxic elements on Earth and are major environmental contaminants in mining wastes.^1^ These elements are highly toxic not only to mammals, but also to plants. One of the reasons for this high toxicity is that Tl mimics the potassium ion (K^+^) and As in the form of arsenate (AsO_4_^3−^) mimics phosphate (PO_4_^3−^).^2,3^ The respective interactions of Tl-K and As-P cause fatal disturbances in many essential processes in plant metabolism. Consequently, most normal plants do not tolerate tissue concentrations >10 mg kg^−1^ Tl or As without suffering major toxicity symptoms. There are, however, rare plants (hyperaccumulators) that have evolved remarkable degrees of tolerance to these toxic elements. Thallium hyperaccumulation is recognized at >100 mg kg^−1^ Tl, while As hyperaccumulation at >1000 mg kg^−1^ As in living shoots.^4^ Hyperaccumulation of Tl is extremely rare and known principally from two species of Brassicaceae: *Biscutella laevigata* (with up to 32 700 mg kg^−1^ in leaves), and *Iberis linifolia* subsp. *intermedia* (syn *I. intermedia*; with up to 4000 mg kg^−1^ in leaves).^5,6^ Arsenic hyperaccumulation is also rare and known mainly from ferns of the genus *Pteris* including *P. vittata* and *Pityrogramma calomelanos*.^7,8^

The abandoned Allchar Mine on Kožuf Mountain in the Republic of North Macedonia is a globally unique deposit with the highest known grades of Tl and As mineralization,^9,10^ and at 500 t its Tl reserves are the greatest in the world.^11^ This former mine is located within an As-Sb-Tl-Hg volcanogenic hydrothermal deposit of several ore bodies in a zone 2 km by 300–500 m wide,^12^ with a highly unusual mineral composition. Apart from Tl minerals (mainly lorandite and vrbaite) and minerals of As, it includes sulfides of Hg and Fe and sulphosalts of Sb and Pb.^9^ The mineralized outcropping soils (as well as the old mine wastes) at the site contain exceptional concentrations of As and Tl with up to 142 g kg^−1^ As and 18 g kg^−1^ Tl, making them possibly the most toxic soils in the world.^13^ The unique mineral and soil composition of the area resulted in a similarly unique flora represented by a considerable number of edaphic endemic species closely associated with a particular substrate type throughout their distribution.^14^ This type of endemism is further favored by the orography, considering the mountainous character of the area. So far, seven endemic taxa have been found near the abandoned Allchar mine: *Centaurea kavadarensis, C. leucomala, Knautia caroli-rechingeri, Onobrychis degenii, Thymus alsarensis, Viola allchariensis*, and *V. arsenica*.^15^ Analysis of metal accumulation in their plant tissues revealed different patterns of As and Tl uptake, with Tl generally accumulated at higher concentrations. Most species (*Centaurea kavadarensis, C. leucomala, Knautia caroli-rechingeri, Onobrychis degenii*) are metal excluders, with the highest metal concentrations in the roots, but well below the hyperaccumulation thresholds. Exclusion as the predominant strategy was also observed in *Thymus alsarensis*, but with foliar Tl concentrations slightly above the threshold of 100 mg kg^−1^. Higher concentrations of As and Tl were found in *Viola* species. Besides two steno-endemic species (*V. allchariensis* and *V. arsenica*), the widely distributed *Viola tricolor* subsp. *macedonica* was also found in the metalliferous area of Allchar. Three *Viola* taxa differ strongly in their metal (hyper)accumulation characteristics: *V. tricolor* subsp. *macedonica* has up to 1.46 mg kg^−1^ As and 4290 mg kg^−1^ Tl in its leaves, *V. allchariensis* has up to 4.26 mg kg^−1^ As and 2190 mg kg^−1^ Tl and *V. arsenica* has 32.3 mg kg^−1^ As and 9090 mg kg^−1^ Tl.^15,16^ Differences were also found in other plant tissues, both below- and above-ground (stems, flowers, seeds). Whereas Tl accumulates mainly in leaves, and to a lesser extent in flowers and seeds, the highest concentrations of As were found in the seeds of *V. allchariensis* and *V. arsenica* (108 and 1390 mg kg^−1^, respectively), followed by the root concentrations of all three species studied (24, 211, and 158 mg kg^−1^ in *V. allchariensis, V. arsenica*, and *V. tricolor* subsp. *macedonica*, respectively).^15^

All three taxa from the Allchar site belong to the *Melanium* Ging. section, one of the most numerous within the genus *Viola*,^17^ which is known for numerous metallophytes that have been intensively studied for their tolerance to extremely Zn-Pb–enriched soils, especially in the western parts of Central Europe.^18–20^  *Viola lutea*, with several metal accumulating subspecies (*V. lutea* subsp. *lutea, V. lutea* subsp. *sudetica, V. lutea* subsp. *calaminaria, V. lutea* subsp. *westfalica*), has been particularly intensively investigated.^18,21,22^ The analysis showed an excluder strategy of *Viola* species at both low- and highly loaded metalliferous sites, with the lowest metal concentrations found in plant shoots. An important role in this exclusion process is attributed to arbuscular mycorrhizal fungi (AMF), which prevent the metal accumulation in photosynthetic plant tissues through complexation or metal compartmentalization in vacuoles or cell walls.^18^ This is particularly true for the endemic zinc violets, *V. lutea* subsp. *calaminaria* and *V. lutea* subsp. *westfalica*, which are heavily colonized with AMF (up to 72.5%), in contrast to the weakly colonized *V. lutea* subsp. *lutea* and *V. lutea* subsp. *sudetica*.^18,22^ A different detoxification pattern was observed in *V. principis*, where a dominant compartmentation of Pb and As was found in the palisade mesophyll.^23^ In contrast to the species from Central Europe, *Viola* spp. from sect. *Melanium* from the Balkan Peninsula, where an ultramafic substrate is frequently represented, are characterized by high tolerance to Ni, especially *V. kopaonikensis, V. elegantula*, and *V. beckiana*, which have been shown to be strong Ni accumulators.^24^ In addition to these European *Viola* accumulators from sect. *Melanium*, Cd hyperaccumulation (1090 mg kg^−1^) was found in *V. baoshanensis*, originating from the Baoshan Zn-Pb mine in China. Although concentrations of Zn and Pb also exceed the notional thresholds (3000 and 1000 mg kg^−1^ for Zn and Pb, respectively),^4^ their concentrations in shoots are lower than in roots, unlike Cd.^25^

Surficial contamination with soil particles on plants growing on metalliferous soils presents a persistent problem for accurate determination of endogenous metal(loid) concentrations in plant material samples.^26–28^ Even intensive cleaning and washing of leaves cannot remove all dust if it is embedded in the waxy cuticle or trichomes, although concentrations of common soil elements such as chromium (Cr), iron (Fe), and titanium (Ti) can be useful to indicate whether soil contamination might be present.^29,30^ Experimental studies on washing plant leaves of various Cu-Co (hyper)accumulator plants from DR Congo showed that foliar concentrations were substantially lower than previously reported and likely due to contamination.^31^ Examination of leaves by micro-X-ray fluorescence analysis (µXRF) and/or scanning electron microscopy with energy dispersive spectroscopy can be used to determine whether surficial contamination is the cause of abnormal elemental concentrations in plant material, and in the case of some species from the DR Congo showed that Cu-Co hyperaccumulation was genuine.^32^ The (potential) issue of soil particulate contamination is especially important at the Allchar site because soil Tl and As concentrations are sometimes extremely high, and hence only minute amounts of particles adhered to the leaves could give rise to very high values in the chemical analysis of these leaves.

The main aim of this study was to establish whether surficial contamination is (partially) responsible for the abnormal concentrations of As and Tl in the *Viola* species from Allchar using synchrotron µXRF analysis.

## Materials and methods

### Handheld XRF spectroscopy

A handheld XRF (Thermo Fisher Scientific Niton XL3t 950 GOLDD+) was used to analyse *Viola* specimens (20 specimens, belonging to three taxa) from the Allchar, deposited in the Serbian Herbaria (BEOU and BEO) and the Paris Herbarium (MNHN). The instrument was used in “Soils Mode” coupled with the “Main filter” at 50 kV aiming to excite K-shell of first row transition elements. Each specimen was placed on top of a pure (99.995%) 2-mm-thick plate of titanium and measured for 30 s, respectively. The raw spectra of each measurement were exported and processed using GeoPIXE 7.5 software.^33,34^ Three steps were applied to each spectrum: (i) fitting a background, (ii) decomposing of each spectrum into many spectra corresponding to possible elements that contribute the spectrum, and (iii) estimating the concentrations of possible elements based on the decomposed spectra by considering the density and thickness of specimen during the estimation process.

### Sample collection and processing for further analysis

**Fig. 1:**
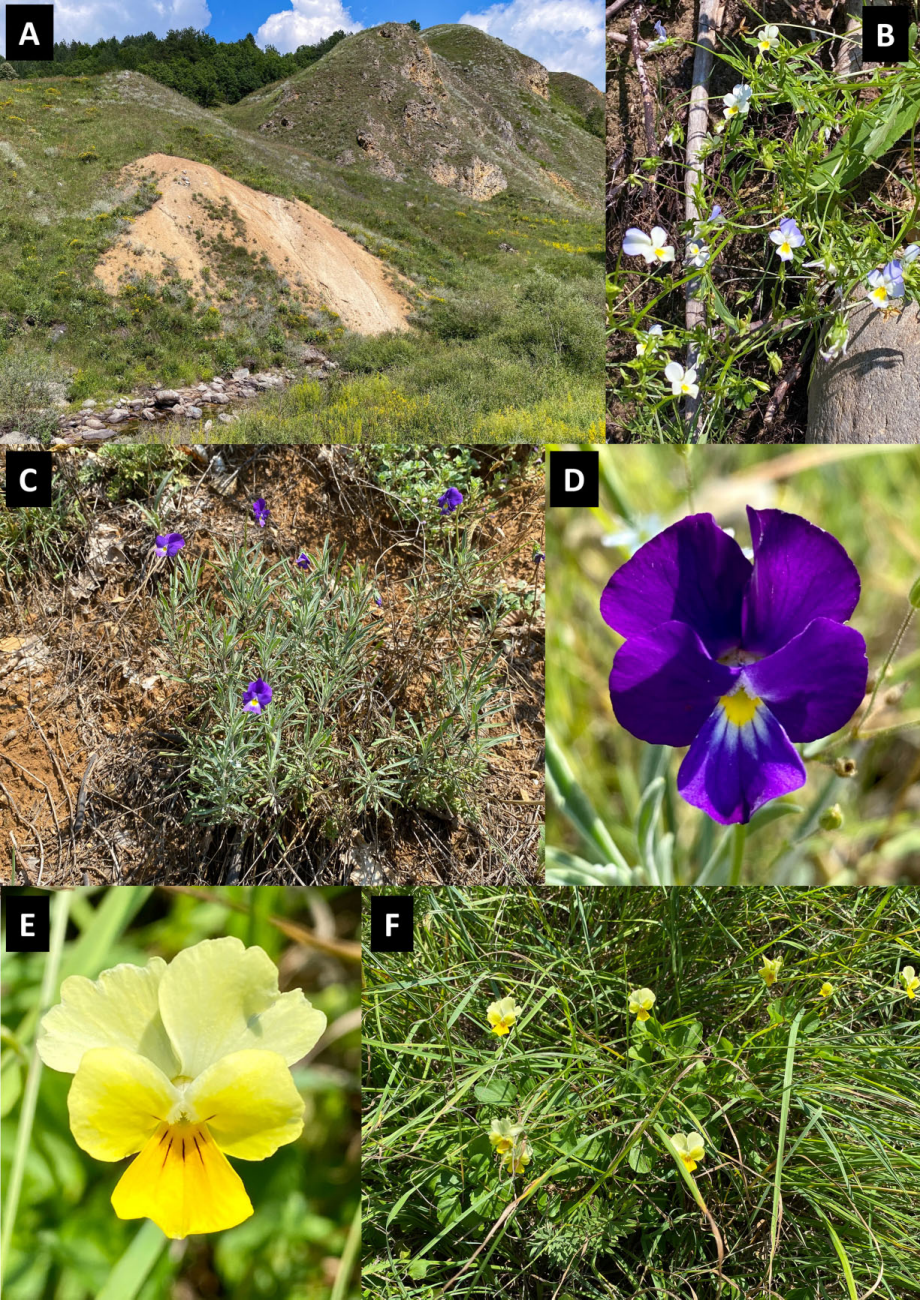
The Allchar mineralized outcrop [panel A], *Viola tricolor* subsp. *macedonica* [panel B], *V. allchariensis* [panels C & D], and *V. arsenica* [panels E & F].

Fieldwork was conducted at the Allchar site and plant tissue samples of *V. tricolor* subsp. *macedonica, V. allchariensis* and *V. arsenica* were collected (Fig. 1). In addition, whole intact shoots were collected, thoroughly washed with water and then rapidly dried between a paper sandwich in a ZIP-lock bag with silica gel for synchrotron µXRF analysis. This method of preparation is not congruent for observing elemental distribution patterns at the scale of cells as elemental redistribution occurred in the sample.^28^ However, we aimed to use synchrotron µXRF analysis to answer the question as to whether the abnormal concentrations of As and Tl genuinely represent hyperaccumulation, or are the result of surficial contamination.

### Elemental analysis and chemical extractions of soil samples

Soil samples were air-dried for 48 h at 40°C, then ground and sieved to 250 µm with zirconium oxide beads. The diethylenetriaminepentaacetic acid–triethanolamine (DTPA–TEA) method following that of Lindsay and Norvell^35^ was used to measure the phytoavailable elements. Briefly, soil samples were individually extracted in a 0.005 M DTPA, 0.1 M TEA and 0.01 M CaCl_2_ solution, with a soil:solution ratio of 1:2 (w/v). Throughout the whole extraction process, the temperature and pH of each extracted sample (in a diluted liquid phase) were kept at 25°C and 7.3, respectively. In addition, phytoavailable elements were also assessed using a CaCl_2_ extraction. Briefly, soil samples were extracted in a 0.01 M CaCl_2_ solution [soil:solution ratio 1:10 (w/v)] agitated for 2 h at a constant 20°C temperature. The resulting solution was then filtered through a 0.45 µm membrane and acidified with nitric acid. The concentrations of each element in the extracted solutions were subsequently measured by inductively coupled plasma–atomic emission spectrometry (ICP–AES; Thermo Scientific iCAP PRO Duo X). The pseudo-total elemental concentrations were obtained by pre-digestion of 0.5 g soil samples with 2 ml HNO_3_/6 ml HCl mixture for 16 h, followed by digestion at 120 min at 105°C using a DigiPREP system (SCP SCIENCE). The resulting solutions were individually made to volume with ultrapure water (50 ml), before filtering using a 0.45 µm membrane. The filtered digestates were analysed with ICP–AES, as described for the soil extractions. The standard soil quality control protocol of the International Soil-Analytical Exchange (ISE) from the Wageningen University (Netherlands) was used for these analyses.

### Elemental analysis of plant samples

Prior the analysis the plant material (separated into roots and leaves) was oven-dried at 60°C for at least 48 h and ground to a fine powder (<200 µm) in an impact mill. After 50 ± 5 mg of the samples were weighed into 15 ml polypropylene tubes, they were pre-digested with a mixture of 1 ml HNO_3_ (70%) and 2 ml H_2_O_2_ (30%) for 16 h and then digested in a block heater (DigiPREP MS, SCP SCIENCE) for 180 min at 95°C. Solutions were then diluted to 10 ml with ultrapure water (Millipore 18.2 MΩ·cm at 25°C) and filtered through 0.45 µm syringe filters before analysis by ICP–AES using a Thermo Scientific iCAP PRO Duo X instrument.

### Synchrotron µXRF experiments

X-ray fluorescence microscopy studies were undertaken at PETRA III (Deutsches Elektronen-Synchrotron DESY), a 6 GeV synchrotron radiation source, specifically at the hard X-ray microprobe experiment undulator beamline P06.^36^ P06 is equipped with a cryogenically cooled double-crystal monochromator with Si(111) crystals. Using different focusing optics, the X-ray beam can be focused down to sub-micron level. An ion chamber upstream of the sample is used to monitor the incoming flux, whereas a 500-µm-thick Si PIPS diode with 19-mm-diameter active area [PD300-500CB, Mirion Technologies (Canberra) GmbH, Germany] located downstream of the sample can be used to record the transmitted X-ray intensity, in order to extract absorption data. Multiple XRF detectors allow for the measurement of X-ray fluorescence data. The incident X-ray energy was 16 keV for the whole experiment. A stack of 50 Beryllium compound refractive lenses (RXOPTICS, Germany) and additional pre-focusing was used to focus the X-ray beam down to 2.1 × 3.6 µm (*h* × *v*), resulting in a flux of about 1.25 × 10^11^ ph/s in the focus. For XRF detection, both a Vortex ME4 SDD (Hitachi High-Tech America, Inc.) in 45° geometry and a prototype 16-element SDD Ardesia detector (800-µm-thick chip with about 324 mm^2^ combined active area for all 16 elements, Politecnico Milano, Italy)^37^ in 315° geometry with Xspress 3 pulse processors were used.

### Data processing and statistical analyses

The handheld XRF data were processed using a universal pipeline in the GeoPIXE analysis package (CSIRO), which utilizes a dynamic analysis algorithm developed for nuclear microprobe techniques and synchrotron-based XRF.^33,34,38^ The algorithm deconvolutes a spectrum into fluorescence components for each element based on an iterative process that involves nonlinear least-squares and linear fitting. The pipeline was modified to process the raw portable XRF data and required two inputs: instrument specifications and sample areal density (mass/area in mg cm^−2^) described elsewhere.^39^ The elemental concentrations in leaf samples are given as means and standard errors. Significant differences between taxa were determined using the Kruskal–Wallis H-test, followed by Dunn's test for pairwise multiple comparisons (*P* < 0.05) using Xlstat software (version 2023.2.0) and indicated by different letters. The same software was used to generate boxplots (mean, minimum, and maximum values in mg kg^−1^) representing As and Tl concentrations in *Viola* taxa analysed). The synchrotron XRF measurements were processed using nonlinear least-squares fitting as implemented in PyMCA.^40^ The fitted results were then calibrated using calibration foils (Micromatter Technologies, Inc.), which produced 32-bit.tiff images with pixel values corresponding to µg cm^−2^ areal density of the element in question. The figures were prepared in ImageJ^41^ by changing the LUT to “Fire,” adjusting of the maximum values and adding concentration bars using the “Calibration Bar” tool and adding length scales.

## Results

### Elemental concentrations in *Viola* taxa

**Fig. 2:**
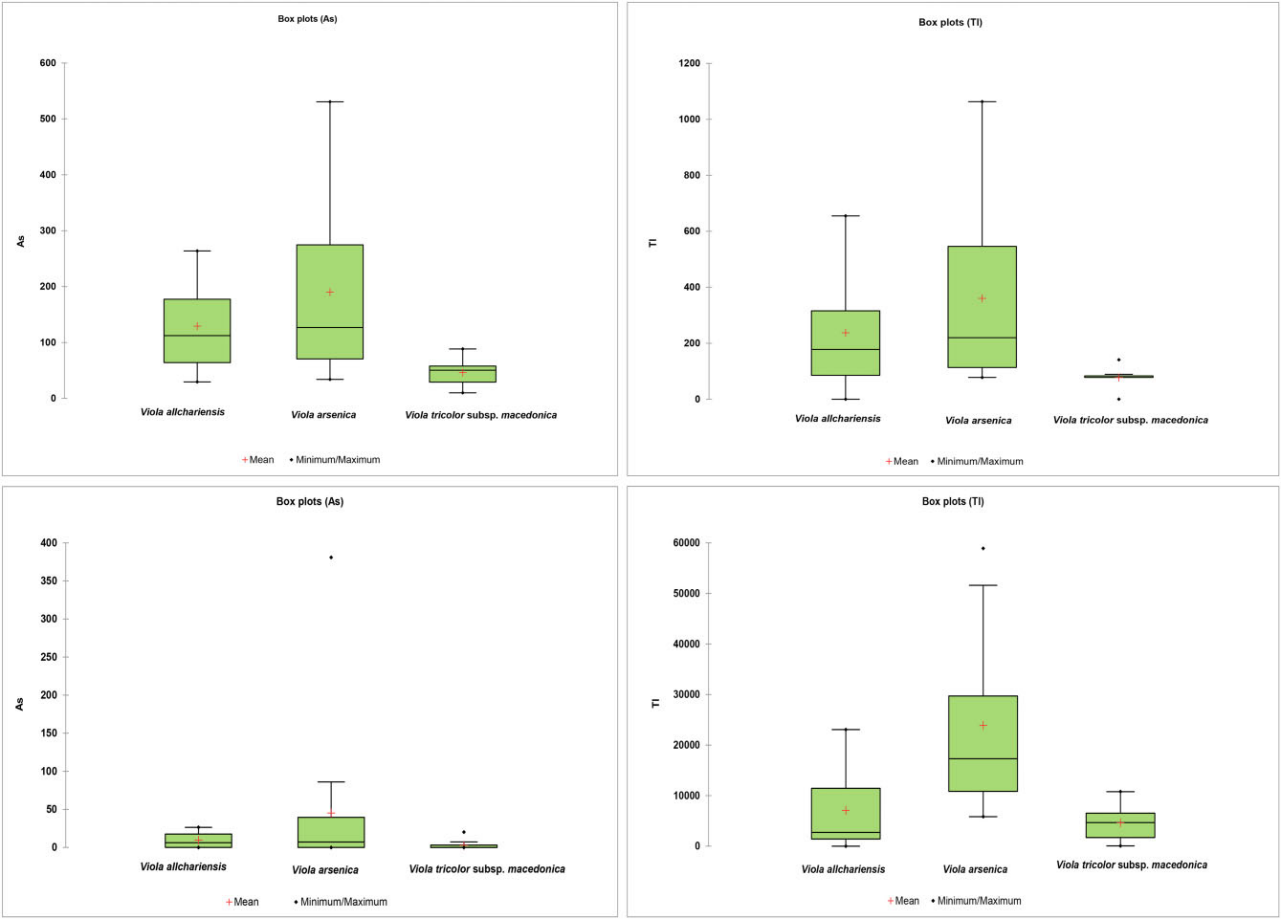
Boxplots of As and Tl concentrations (in mg kg^−1^) in the leaves of *Viola allchariensis, V. arsenica* and *V. tricolor* subsp. *macedonica* from **A**. herbarium and **B**. field samples.

The results of the XRF scanning of herbarium specimens show significant variations in As and Tl concentrations between the taxa. The highest concentrations of As were found in the leaves of *V. allchariensis* (540 mg kg^−1^) from BEO. In the specimens from BEOU and MNHN, these concentrations were much lower, ranging from 29‒264 mg kg^−1^ to 119‒304 mg kg^−1^, respectively. Arsenic concentrations in *V. arsenica* from BEOU were as high as 531 mg kg^−1^, whereas these concentrations in *V. tricolor* subsp. *macedonica* were much lower, ranging from 10 to 88 mg kg^−1^. Thallium concentrations determined by XRF analysis were higher than As concentrations, with 1060 mg kg^−1^ being highest value in *V. arsenica* leaves (Fig. 2a). Slightly lower concentrations were found in *V. allchariensis*, up to 655 mg kg^−1^, whereas in *V. tricolor* subsp. *macedonica* they ranged from 78 to 141 mg kg^−1^.

In the leaves of all three *Viola* taxa collected in the field Tl concentrations were extremely high, the highest being found in *V. arsenica*. The mean concentration was 23 900 mg kg^−1^, whereas the

highest individual had up to 58 900 mg kg^−1^ (Table 1; Fig. 2b). For *V. allchariensis* and *V. tricolor* subsp. *macedonica* these concentrations were slightly lower, but still well above the hyperaccumulation threshold, with the maximum concentration being 23 100 and 10 800 mg kg^−1^, respectively. Very variable concentrations were also found for As, with the highest individual concentration in *V. arsenica* (381 mg kg^−1^), but without exceeding the hyperaccumulation threshold. In *V. allchariensis* these concentrations were up to 26 mg kg^−1^, and in *V. tricolor* subsp. *macedonica* even lower (only up to 20 mg kg^−1^). Concentrations of macro- and microelements other than As and Tl were unremarkable (Table 1). Arsenic and thallium, which were found in elevated concentrations in the leaf samples of the three studied *Viola* taxa, showed even higher levels in the soil. Thus, the total concentrations of As in the studied samples reached 33 500 mg kg^−1^, whereas the concentrations of Tl were up to 4430 mg kg^−1^. The extractable concentrations of these elements were much lower, up to 7 and 15 mg kg^−1^ for As and Tl, respectively.

**Table 1.** Elemental concentrations in field collected leaf samples of *Viola allchariensis, V. arsenica*, and *V. tricolor* subsp. *macedonica* from the Allchar site, given as means and standard errors, analysed by ICP–AES^a^

	*Viola allchariensis*	*Viola arsenica*	*Viola tricolor* subsp*. macedonica*
Al	54.4 ± 7.94 a	56.1 ± 11.4 a	66.1 ± 15 a
As	9.57 ± 2.91 a	45.2 ± 28.5 a	2.74 ± 1.45 a
Ba	18.4 ± 9.77 a	11.1 ± 3.59 a	53 ± 14.7 a
Ca	16 100 ± 2170 ab	11 600 ± 1480 a	17 000 ± 1490 b
Cu	0.01 ± 0 a	3.02 ± 2.28 a	1.51 ± 0.607 a
Fe	157 ± 29.9 a	134 ± 23.9 a	164 ± 25.5 a
K	17 200 ± 1280 a	25 300 ± 2150 b	26 100 ± 1650 b
Mg	4040 ± 479 a	5290 ± 649 ab	5580 ± 532 b
Mn	131 ± 17.7 b	44.5 ± 12.6 a	61.7 ± 10.4 a
Na	13 ± 3.31 a	40.9 ± 18.4 a	110 ± 59.8 a
P	2050 ± 311 a	3100 ± 294 b	2730 ± 283 ab
S	3290 ± 293 a	2870 ± 155 a	3580 ± 457 a
Sb	3.73 ± 0.445 ab	2.64 ± 0.456 a	8.6 ± 1.98 b
Se	4.64 ± 0.598 ab	3.08 ± 0.617 a	9.68 ± 2.09 b
Tl	7060 ± 2240 a	23 900 ± 5150 b	4650 ± 853 a
Zn	53.2 ± 5.74 a	41.8 ± 5.02 a	108 ± 8.79 b

^a^Different letters indicate significant differences between the plant samples according to the Kruskal–Wallis ANOVA, followed by Dunn's post hoc test.

### Broad-scale patterns of elemental distribution in *Viola* specimens

**Fig. 3:**
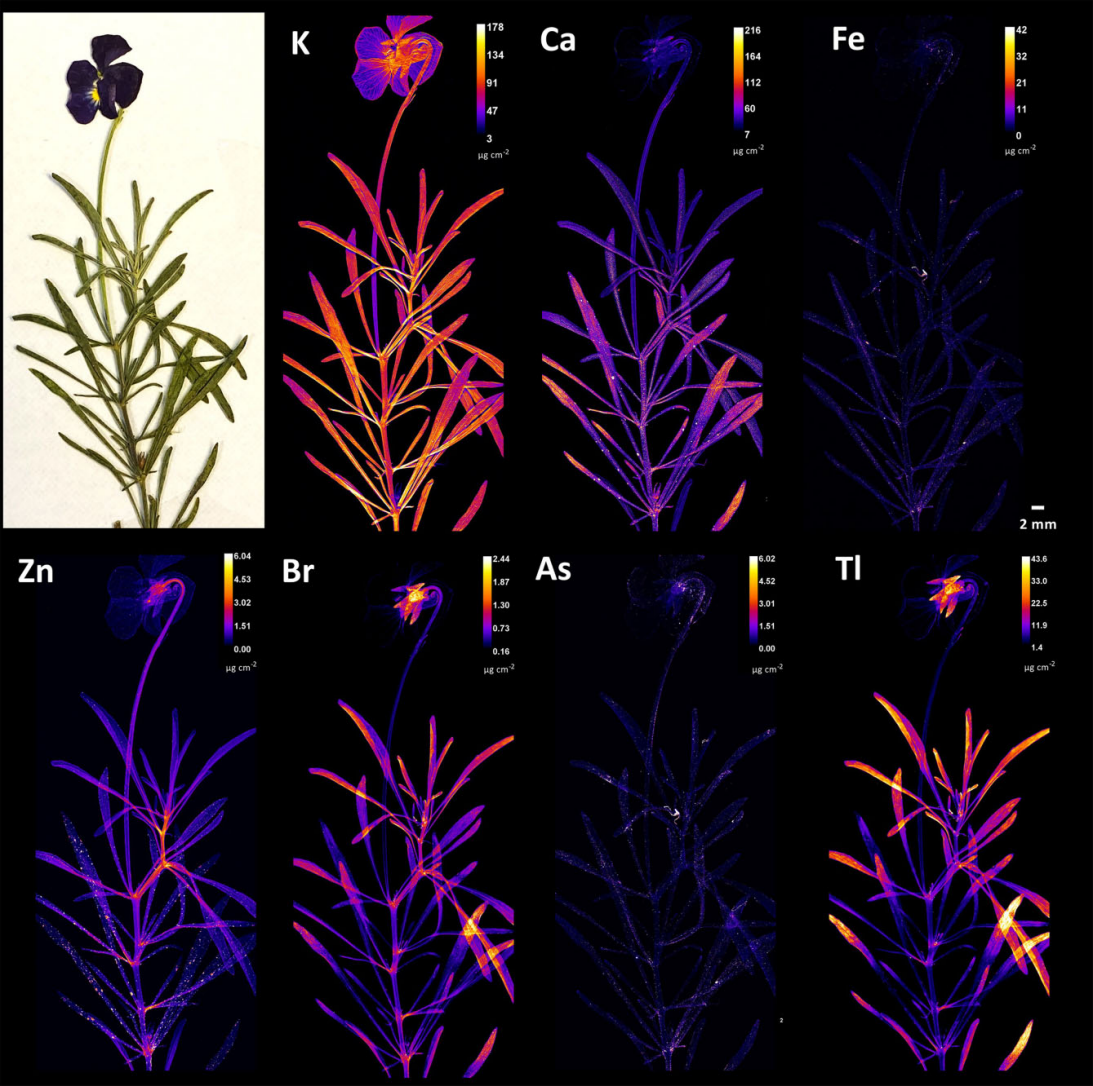
Synchrotron µXRF elemental maps showing the distributions of K, Ca, Fe, Zn, Br, As and Tl in a shoot of *Viola allchariensis.*

**Fig. 4:**
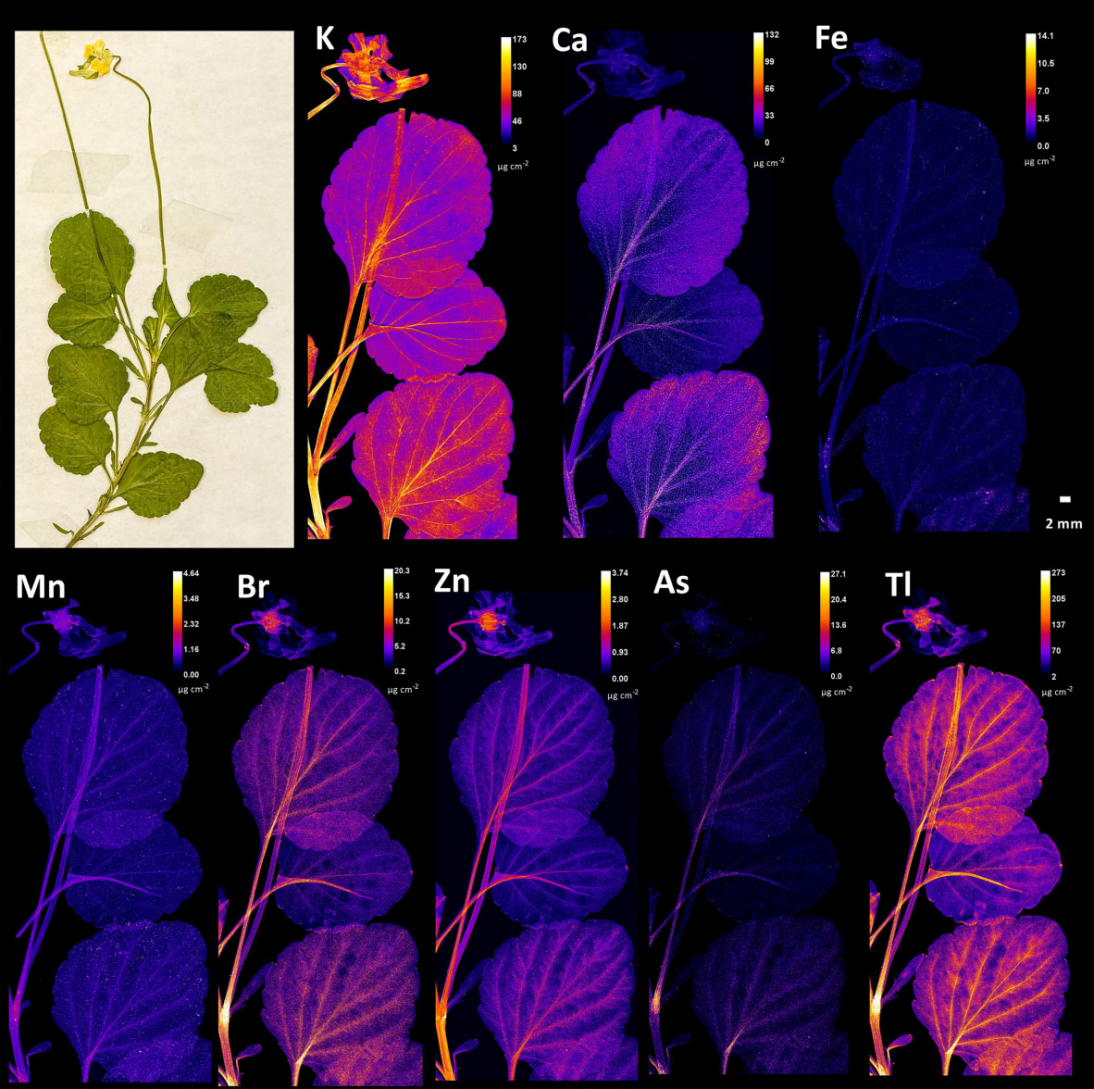
Synchrotron µXRF elemental maps showing the distributions of K, Ca, Fe, Mn, Br, Zn, As and Tl in a shoot of *Viola arsenica*.

**Fig. 5:**
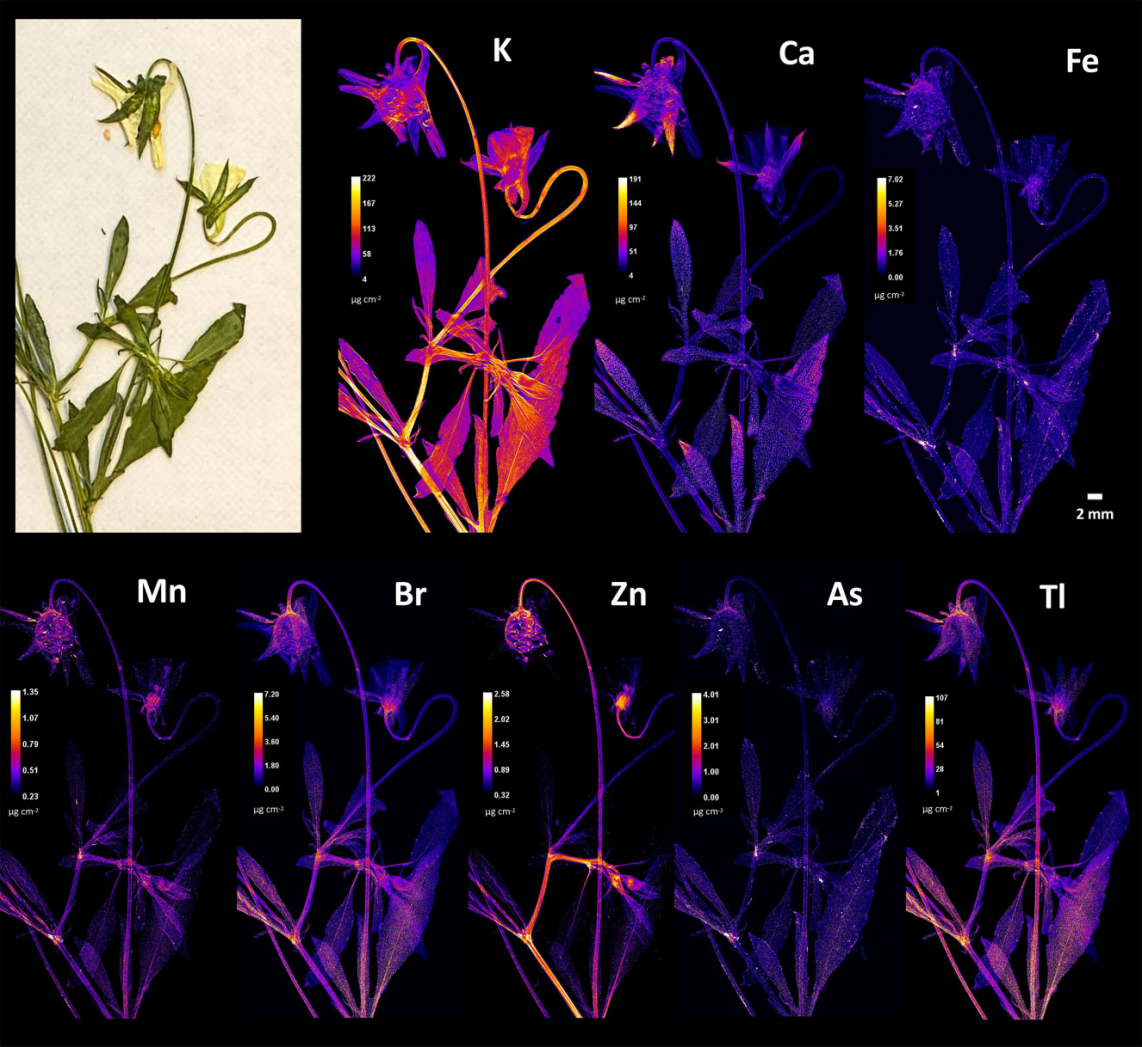
Synchrotron µXRF elemental maps showing the distributions of K, Ca, Fe, Mn, Br, Zn, As, and Tl in a shoot of *Viola tricolor* subsp. *macedonica.*

**Fig. 6:**
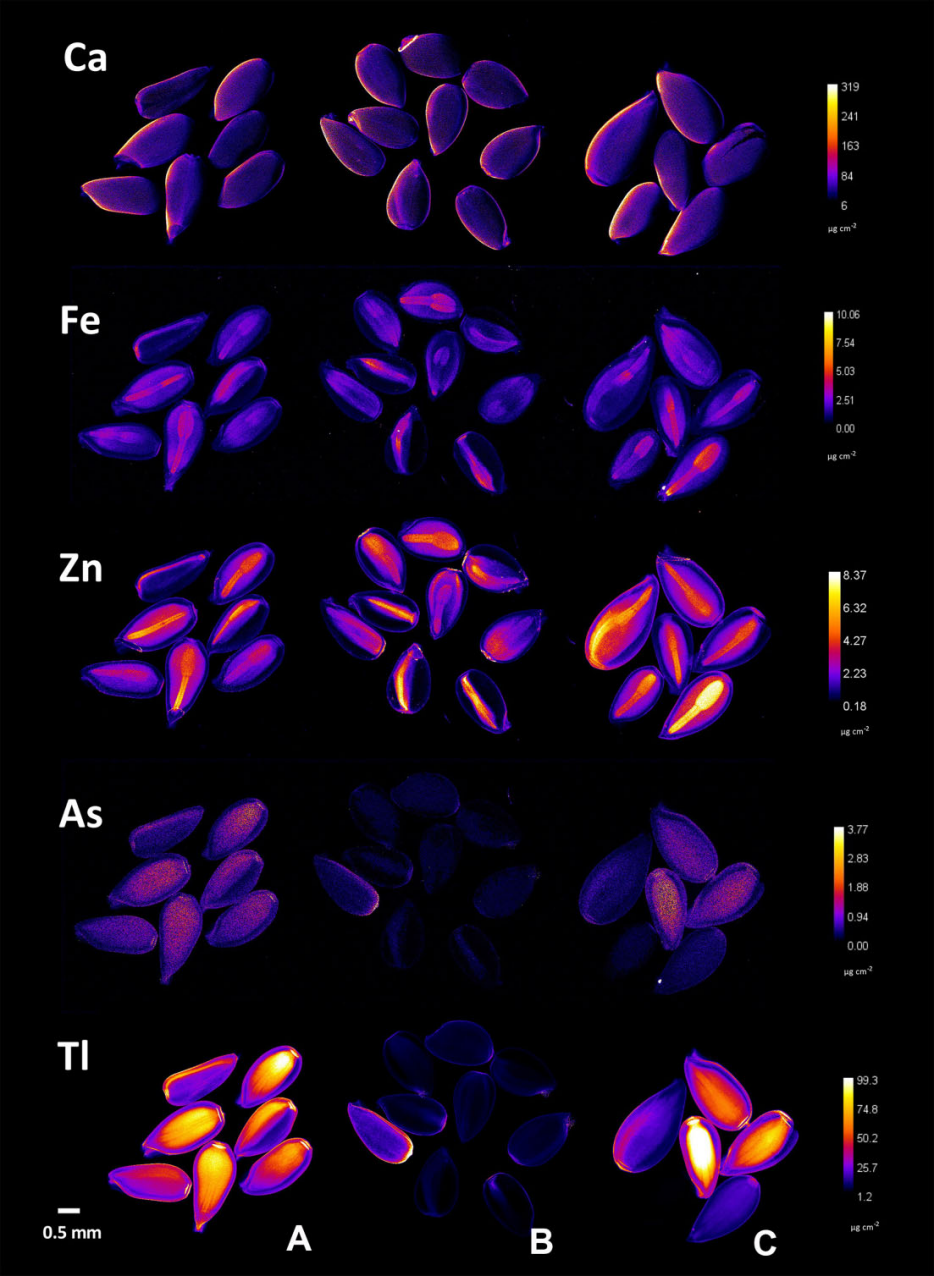
Synchrotron µXRF elemental maps showing the distributions of Ca, Fe, Zn, As, and Tl in seeds of **A**. *V. allchariensis*, **B**. *V. tricolor* subsp*. macedonica* and **C**. *V. arsenica.*

**Fig. 7:**
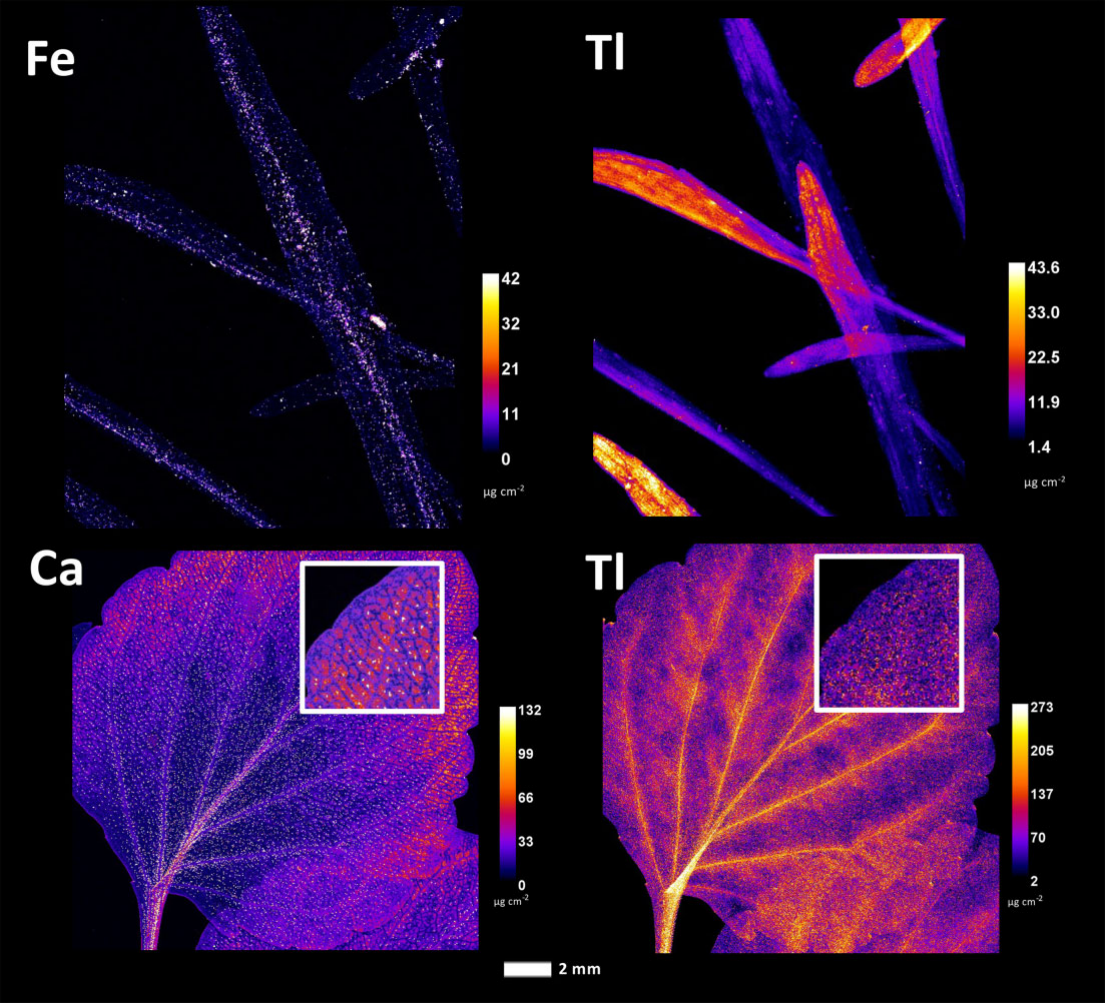
Synchrotron µXRF elemental maps showing the distributions of Fe, Ca, and Tl in *Viola allchariensis* [top] and *V. arsenica* [bottom].

**Fig. 8:**
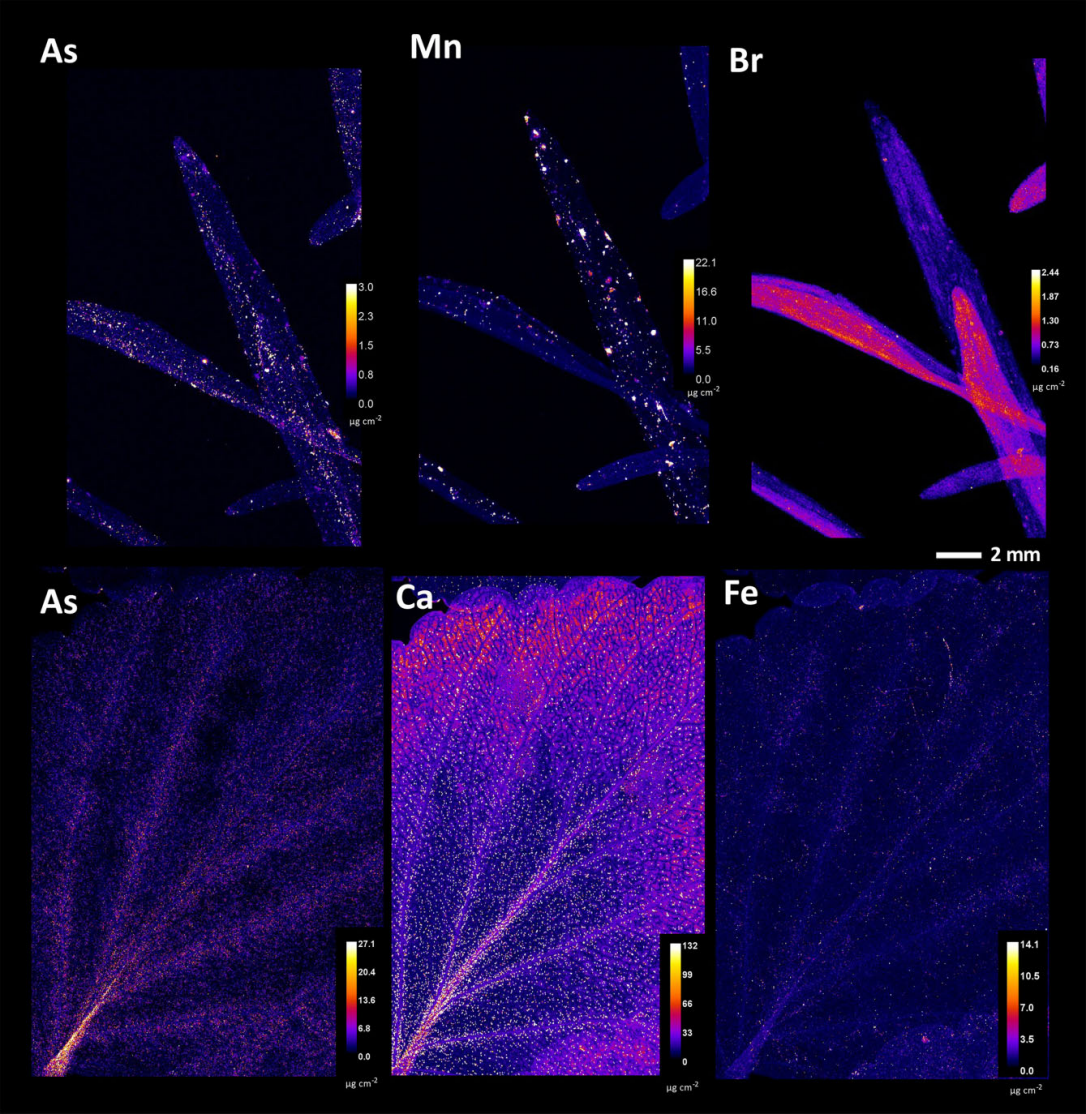
Synchrotron µXRF elemental maps showing the distributions of As, Mn, Br, Ca, and Fe in *Viola allchariensis* [top] and *V. arsenica* [bottom].

Elemental distribution within the studied *Viola* taxa is shown in Figs. 3–8. Significant differences were observed in the distribution patterns of the various elements. X-ray fluorescence microscopy showed a predominant localization of Tl in the leaf apices, which was much more intense in the upper parts of *V. allchariensis* (Fig. 3). This element was also enriched in the sepals, particularly in the lower parts and at their junction with the pedicel. The same was found for Br, but with a slightly less intense accumulation in the leaves and more pronounced at the base of the sepals. In the stems, the basal part of the leaves and the petals, the amounts of these elements were quite low. Potassium was almost evenly distributed throughout the plant, with peaks in the lower part of the plant, in the stem and in the leaves. It was particularly enriched in the midribs and basal parts of the leaves. In addition to the sepals, enrichment of K was also observed in the veins or “pencil lines” of the petals and in the petal margins of *V. allchariensis*. Calcium was enriched in the upper parts of the leaves but, unlike Tl and Br, only in the lower part of the plants. However, it should be noted that light elements, such as K and Ca, are affected by self-absorption effects, meaning that the “probe depth” is limited with signal originates mainly from the top 100 µm, whereas for heavier elements (>Zn) it originates from the full depth of the tissue. The distribution of Zn is homogeneous, but in lower concentrations than those of most the other elements analysed. The highest concentrations were found in the sepals and pedicels, but also in the nodes and petioles. Both As and Fe were under-represented in the plant tissues of *V. allchariensis*.

The pattern of elemental distribution in *V. arsenica* is similar to that in *V. allchariensis*. The most concentrated element was potassium, with a homogeneous distribution and peaks of concentration in the stems, petioles, and leaf veins and veinlets. It was also enriched in the flower (petals and sepals) and in the pedicel (Fig. 4). A similar pattern was observed for Tl, Br, Zn, and As, but with lower elemental concentrations, especially for Zn and As (Tl > Br > Zn > As). Whereas concentrations of Tl, Br, and Zn were observed in the flowers, especially in their pistils, this was not the case for As. Calcium, manganese, and iron were evenly distributed within the plant, with slightly higher Ca concentrations compared to the other two elements.

X-ray fluorescence microscopy of *V. tricolor* subsp. *macedonica* plant tissues revealed substantial differences between this taxon and the other two *Viola* species from Allchar. Most elements were enriched in stems and petioles, both distally and proximally (Fig. 5). This trend was also observed for K, the most concentrated of all the elements analysed. In addition to the stems and petioles, the highest concentrations were found in the leaves, their midribs and veins, but also in a lamina. High K content was also found in the petals and their veins, but less pronounced than Ca, which was generally evenly distributed in the plant tissues of *V. tricolor* subsp. *macedonica*, with the highest concentrations found in the sepals. Most of the trace elements analysed (Tl, Zn, Br, and Mn) were also enriched in the flowers, in their basal parts, pistils, and sepals. They were also found in the stems and at peak levels in the nodes. Several elements analysed, such as As, Se, Fe, and Hg, were homogeneously distributed in the plant tissues, but at much lower concentrations.

The elemental distribution in the seeds of the three *Viola* taxa studied follows the pattern observed for other plant tissues, considering the similarities between *V. allchariensis* and *V. arsenica* (Fig. 6). This is especially true for the distribution of Tl and As, which are enriched in the endosperm, and in the case of Tl, in much higher concentrations. The concentrations of these elements are much lower in the seeds of *V. tricolor* subsp. *macedonica*. The distribution of Ca was homogeneous in all three taxa, with a predominant enrichment in the seed coat. Slight differences were observed for Fe and Zn, both of which were enriched in the embryos, but at the highest concentrations in *V. arsenica*.

To exclude surficial contamination as a cause of enrichment in the synchrotron µXRF elemental maps, the distribution of Tl, an element of particular interest to *Viola* taxa from Allchar, was compared with that of Fe and Ca, macroelements present in soil at very high concentrations (Fig. 7). Both Fe and Ca elemental maps showed high concentrations of particulates, uniformly distributed over the leaf blades of the *Viola* taxa studied. The accumulation of Tl showed a markedly different pattern in both species. Whereas in *V. allchariensis* it was seen as hotspots in the upper part of the leaves, in *V. arsenica* the highest concentration was found in the leaf midrib, veins, and veinlets. As for As distribution, the hotspots in *V. tricolor* subsp. *macedonica* and *V. allcharensis* co-locate with those of Fe and Mn (Figs. 5 and 8). In *V. arsenica* this pattern of As enrichment is somewhat different and coincides with Ca (oxalate) deposition and Br hotspots, but not with Fe hotspots.

## Discussion

The XRF analysis of herbarium specimens of two endemic *Viola* species, *V. allchariensis* and *V. arsenica*, and the widely distributed *V. tricolor* subsp. *macedonica* showed increased As concentrations in leaf tissues, but without reaching the hyperaccumulation threshold. Although striking differences were found both between and within the same taxa, the mean values of the XRF measurements for As in *V. allchariensis* and *V. arsenica* were approximately the same (∼180 mg kg^−1^). In *V. tricolor* subsp. *macedonica* the mean As concentration was several times lower. The herbarium specimens of these taxa also had the lowest mean and absolute Tl concentrations, although the hyperaccumulation threshold was reached in one of them. In *V. allchariensis* and *V. arsenica*, Tl concentrations were well above 100 mg kg^−1^, especially in *V. arsenica*, where almost all specimens showed hyperaccumulation of this element (Fig. 2a).

Compared to the concentrations determined by XRF analyses, the results of chemical analysis showed significantly lower values of As for all three *Viola* taxa studied, both in mean and in absolute terms. This was especially true for *V. allchariensis* and *V. tricolor* subsp. *macedonica*. For *V. arsenica*, these differences were somewhat smaller, in part due to strikingly higher concentrations in individual specimens. This could indicate both individual differences, which are common at mine tailing sites, and possible contamination, which is very difficult to rule out absolutely, regardless of sample processing. With the exception of the highest As concentration in the leaf tissue of *V. allchariensis*, the mean and highest concentrations of As and Tl were higher in all three *Viola* taxa analysed in this study than in that by Bačeva Andonovska *et al*.^15^ Whereas As concentrations were well below the threshold for hyperaccumulation in all individuals (<1000 mg kg^−1^), hyperaccumulation of Tl was observed in all samples. The highest Tl concentrations were found in *V. arsenica*, where hyperaccumulation was detected in every individual (Fig. 2b), with a maximum value that exceeded those previously found in some of the strongest hyperaccumulators of this element—*Biscutel lalaevigata* (up to 32 700 mg kg^−1^), *Iberis linifolia* subsp. *intermedia* (up to 400 mg kg^−1^), and *Silene latifolia* (up to 1500 mg kg^−1^).^42^

The results of this study show significant differences in the uptake of As and Tl as dominant elements in the Allchar area. Although the pseudo-total concentration of soil As was significantly higher than that of Tl, there were no significant differences between their extractable concentrations (all <10 mg kg^−1^). However, considering that Tl concentrations in the leaves are several-fold higher than those of As, this suggests plant affinity as a determining factor for accumulation. Another possibility for the extreme metal concentrations in the leaves could be surficial contamination with soil particles, which is especially problematic at metalliferous sites with extremely high concentrations of suspected hyperaccumulated elements, in this case As and Tl. However, the results show that Tl is indeed endogenous in the *Viola* taxa, considering the markedly different distribution patterns in all taxa analysed, and the distribution patterns of macroelements, such as Fe and Ca, present in the soil at very high concentrations (Fig. 7). In contrast, As appears to be affected by the contamination. The coincidence of As hotspots in *V. tricolor* subsp. *macedonica* and *V. allcharensis* with those of Fe and Mn, most likely of soil origin, suggests contamination as the source of As enrichment (Figs. 5 and 8). However, As appears to be endogenous in *V. arsenica*, considering a different distribution pattern consistent with Ca deposition and Br hotspots.

This study aimed to assess the potential hyperaccumulation of As and Tl, the most abundant trace elements in Allchar mine area, in leaves of three *Viola* taxa. Hyperaccumulation level concentrations of Tl were found in all taxa studied, with up to 58 900 mg kg^−1^ in the sample of *V. arsenica*, which is the highest known concentration of this element in plant above-ground tissues. Compared to previous studies,^15^ not only were significantly higher Tl concentrations found in the *Viola* species studied, but synchrotron µXRF analysis was performed to determine whether the As and Tl concentrations were endogenous or the result of contamination. Although dehydrated shoots are not optimal for this type of analysis, the results showed that As is strongly influenced by contamination in *V. tricolor* subsp. *macedonica* and *V. allcharensis*, but not in *V. arsenica*, where it appears to be endogenous, which is also true for Tl in all three taxa. The results of this study not only provide information on the distribution of As and Tl in the taxa studied, but also form the basis for more detailed studies under controlled conditions, including plant dosing experiments.

## Data Availability

The data that support this study will be shared upon reasonable request to the corresponding author.

## References

[1] Lennartson A. , Toxic Thallium, Nature Chem., 2015, 7 (7), 610. 10.1038/nchem.2286 26100812

[2] DiTusa S. F., Fontenot E. B., Wallace R. W., Silvers M. A., Steele T. N., Elnagar A. H., Dearman K. M., Smith A. P., A Member of the Phosphate Transporter 1 (Pht1) Family from the Arsenic-Hyperaccumulating Fern *Pteris vittata* Is a High-Affinity Arsenate Transporter, New Phytol., 2016, 209 (2), 762–772. 10.1111/nph.13472 26010225

[3] Rader S. T., Maier R. M., Barton M. D., Mazdab F. K., Uptake and Fractionation of Thallium by *Brassica juncea* in a Geogenic Thallium-Amended Substrate, Environ. Sci. Technol., 2019, 53 (5), 2441–2449. 10.1021/acs.est.8b06222 30707569 PMC7029784

[4] van der Ent A., Baker A. J. M., Reeves R. D., Pollard A. J., Schat H., Hyperaccumulators of Metal and Metalloid Trace Elements: Facts and Fiction, Plant Soil, 2013, 362 (1-2), 319–334. 10.1007/s11104-012-1287-3

[5] LaCoste C., Robinson B., Brooks R., Anderson C., Chiarucci A., Leblanc M., The Phytoremediation Potential of Thallium-Contaminated Soils Using *Iberis* and *Biscutella* Species, Int. J. Phytorem., 1999, 1 (4), 327–338. 10.1080/15226519908500023

[6] Fellet G., Pošćić F., Casolo V., Marchiol L., Metallophytes and Thallium Hyperaccumulation at the Former Raibl Lead/Zinc Mining Site (Julian Alps, Italy), Plant Biosyst., 2012, 146 (4), 1023–1036. 10.1080/11263504.2012.703250

[7] Campos N. V., Arcanjo-Silva S., Freitas-Silva L., de Araújo T. O., Souza-Fernandes D. P., Azevedo A. A., Arsenic Hyperaccumulation in *Pityrogramma calomelanos* L. (Link): Adaptive Traits to Deal with High Metalloid Concentrations, Environ. Sci. Pollut. Res., 2018, 25 (11), 10720–10729. 10.1007/s11356-017-1085-9 29396820

[8] Xie Q.-E., Yan X.-L., Liao X.-Y., Li X., The Arsenic Hyperaccumulator Fern *Pteris vittata* L., Environ. Sci. Technol., 2009, 43 (22), 8488–8495. 10.1021/es9014647 20028042

[9] Volkov A. V., Serafimovski T., Kochneva N. T., Tomson I. N., Tasev G., The Alshar Epithermal Au-As-Sb-Tl Deposit, Southern Macedonia, Geol. Ore Deposits, 2006, 48 (3), 175. 10.1134/S1075701506030020

[10] Boev B., Jovanovski G., Makreski P., Geology and Mineralogy of Allchar Sb-As-Tl-Au Deposit. II. Congress of Geologists of Republic of Macedonia, Geol. Maced., 2012, 3, 215–232.

[11] Ivanov T. , Allchar the Richest Ore Deposit of Tl in the World, Proceedings of the Workshop on the Feasibility of the Solar Neutrino Detection with Pb by Geochemical and Accelerator Mass Spectrometry, GSI-Report 86–9, Munich, 1986.

[12] Janković S. R. , Metallogenic Features of the Alsar Sb-As-Tl-Au Deposit, Neues Jb. Miner. Abh., 1993, 166, 25‒41.

[13] Đorđević T., Drahota P., Kolitsch U., Majzlan J., Peřestá M., Kiefer S., Stöger-Pollach M., Tepe N., Hofmann T., Mikuš T., Tasev G., Synergetic Tl and As Retention in Secondary Minerals: an Example of Extreme Arsenic and Thallium pollution, Appl. Geochem., 2021, 135, 105114. 10.1016/j.apgeochem.2021.105114

[14] Whittaker R. H. , The Ecology of Serpentine Soils, Ecology, 1954, 35 (2), 258. 10.2307/1931126

[15] Bačeva Andonovska K., Stafilov T., Matevski V., Accumulation Abilities of Endemic Plant Species from the Vicinity of an As-Sb-Tl Abandoned Mine, Allchar, Kožuf Mountain, In: Balabanova B., Stafilov T. (eds), Contaminant Levels and Ecological Effects. Springer, Cham, Switzerland 2021, pp 375‒402. 10.1007/978-3-030-66135-9_13

[16] Bačeva K., Stafilov T., Matevski V., Bioaccumulation of Heavy Metals by Endemic *Viola* Species from the Soil in the Vicinity of the As-Sb-Tl Mine “Allchar”, Republic of Macedonia, Int. J. Phytorem., 2014, 16 (4), 347. 10.1080/15226514.2013.783551 24912236

[17] Marcussen T., Ballard H. E., Danihelka J., Flores A. R., Nicola M. V., Watson J. M., A Revised Phylogenetic Classification for *Viola* (Violaceae), Plants, 2022, 11 (17), 2224. 10.3390/plants11172224 36079606 PMC9460890

[18] Słomka A., Kuta E., Szarek-Łukaszewska G., Godzik B., Kapusta P., Tylko G., Bothe H., Violets of the Section *Melanium*, Their Colonization by Arbuscular Mycorrhizal Fungi and Their Occurrence on Heavy Metal Heaps, J. Plant Physiol., 2011, 168 (11), 1191. 10.1016/j.jplph.2011.01.033 21492955

[19] Słomka A., Gubernat M., Pliszko A., Bothe H., The Unusual Property of the Sand Violet, *Viola rupestris*, to Cope with Heavy Metal Toxicity, Flora, 2020, 271, 151663. 10.1016/j.flora.2020.151663

[20] Bothe H., Vogel-Mikuš K., Pongrac P., Likar M., Stepic N., Pelikon P., Vavpetič P., Jeromel L., Regvar M., Metallophyte Status of Violets of the Section *Melanium*, Chemosphere, 2013, 93 (9), 1844. 10.1016/j.chemosphere.2013.06.039 23859423

[21] Jedrzejczyk M., Rostanski A., Malkowski E., Accumulation of Zinc and Lead in Selected Taxa of the Genus *Viola* L., Acta Biol. Crac. Ser. Bot., 2002, 44, 49‒55.

[22] Hildebrandt U., Hoef-Emden K., Backhausen S., Bothe H., Bożek M., Siuta A., Kuta E., The Rare, Endemic Zinc Violets of Central Europe Originate from *Viola lutea* Huds., Plant Syst. Evol., 2006, 257 (3-4), 205. 10.1007/s00606-005-0387-4

[23] Lei M., Chen T. B., Huang Z. C., Wang Y. D., Huang Y. Y., Simultaneous Compartmentalization of Lead and Arsenic in Co-Hyperaccumulator *Viola principis* H. de Boiss.: an Application of SRXRF Microprobe, Chemosphere, 2008, 72 (10), 1491. 10.1016/j.chemosphere.2008.04.084 18571691

[24] Tomović G., Đurović S., Buzurović U., Niketić M., Milanović Đ., Mihailović N., Jakovljević K., Accumulation of Potentially Toxic Elements in *Viola* L. (Sect. *Melanium* Ging.) from the Ultramafic and Non-Ultramafic Soils of the Balkan Peninsula, Water Air Soil Pollut., 2021, 232 (2), 1‒8. 10.1007/s11270-021-04992-w

[25] Wu C., Liao B., Wang S. L., Zhang J., Li J. T., Pb and Zn Accumulation in a Cd-Hyperaccumulator (*Viola baoshanensis*), Int. J. Phytorem., 2010, 12 (6), 574. 10.1080/15226510903353195 21166282

[26] van der Ent A., Echevarria G., Pollard A. J., Erskine P. D., X-Ray Fluorescence Ionomics of Herbarium Collections, Sci. Rep., 2019, 9 (1), 4746. 10.1038/s41598-019-40050-6 30894553 PMC6426943

[27] van der Ent A., Purwadi I., Harris H. H., Kopittke P. M., Przybyłowicz W. J., Mesjasz-Przybyłowicz J., Methods for Elucidating Elemental Distribution in Hyperaccumulator Plants, In: van der Ent A., Baker A. J. M., Echevarria G., Simonnot M-O., Morel J. L. (eds), Agromining: Farming for Metals, 2nd edn. Springer, Cham, 2021, pp 197–214.

[28] van der Ent A., Casey L. W., Imam Purwadi I., Erskine P. D., Laboratory µ-X-Ray Fluorescence Elemental Mapping of Herbarium Specimens for Hyperaccumulator Studies, Plant Soil, 2023, (In Press) 10.1007/s11104-023-06201-5.

[29] Reeves R. D., Kruckeberg A. R., Re-Examination of the Elemental Composition of some *Caryophyllaceae* on North American Ultramafic Soils, Ecol. Res., 2018, 33 (4), 715–722. 10.1007/s11284-017-1556-y

[30] Paul A. D. L., van der Ent A., Erskine P. D., Scandium Biogeochemistry at the Lucknow Laterite Plateau, Queensland, Australia, J. Geochem. Explor., 2019, 204, 74–82. 10.1016/j.gexplo.2019.05.005

[31] Faucon M.-P., Shutcha M. N., Meerts P., Revisiting Copper and Cobalt Concentrations in Supposed Hyperaccumulators from SC Africa: Influence of Washing and Metal Concentrations in Soil, Plant Soil, 2007, 301 (1-2), 29–36. 10.1007/s11104-007-9405-3

[32] van der Ent A., Erskine P. D., Malaisse F., Mesjasz-Przybylowicz J., Przybylowicz W. J., Barnabas A. D., Sosnicka M., Harris H. H., Abnormal Concentrations of Cu-Co in *Haumaniastrum katangense, Haumaniastrum robertii* and *Aeolanthus biformifolius*: Contamination or Hyperaccumulation?, Metallomics, 2019, 11 (3), 586–596. 10.1039/c8mt00300a 30664146

[33] Ryan C. G. , Quantitative Trace Element Imaging Using PIXE and the Nuclear Microprobe, Int. J. Imaging Syst. Tech., 2000, 11 (4), 219. 10.1002/ima.1007

[34] Ryan C. G., Etschmann B. E., Vogt S., Maser J., Harland C. L., van Achterbergh E., Legnini D., Nuclear Microprobe—Synchrotron Synergy: towards Integrated Quantitative Real-Time Elemental Imaging Using PIXE and SXRF, Nucl. Instrum. Methods Phys. Res. Sect. B, 2005, 231 (1-4), 183. 10.1016/j.nimb.2005.01.054

[35] Lindsay W. L., Norvell W., Development of a DTPA Soil Test for Zinc, Iron, Manganese, and Copper, Soil Sci. Soc. Am. J., 1978, 42 (3), 421. 10.2136/sssaj1978.03615995004200030009x

[36] Boesenberg U., Ryan C. G., Kirkham R., Siddons P., Alfeld M., Garrevoet J., Núñez T., Claussen T., Kracht T., Falkenberg G., Fast X-ray Micro Fluorescence Imaging with Sub-Micrometer Resolution Integrating a Maia Detector at Beamline P06 at PETRA III, J. Synchrotron Rad., 2016, 23 (6), 1550–1560. 10.1107/S1600577516015289 27787262

[37] Utica G., Fabbrica E., Carminati M., Borghi G., Zorzi N., Ficorella F., Picciotto A., Allegretta I., Falkenberg G., Fiorini C., ARDESIA-16: a 16-Channel SDD-Based Spectrometer for Energy Dispersive X-Ray Fluorescence Spectroscopy, J. Inst., 2021, 16 (7), P07057. 10.1088/1748-0221/16/07/P07057

[38] Ryan C. G., Laird J. S., Fisher L. A., Kirkham R., Moorhead G. F., Improved Dynamic Analysis Method for Quantitative PIXE and SXRF Element Imaging of Complex Materials, Nucl. Instrum. Methods Phys. Res. Sect. B, 2015, 363, 42–47. 10.1016/j.nimb.2015.08.021

[39] Purwadi I., Casey L. W., Ryan C. G., Erskine P. D., van der Ent A., X-Ray Fluorescence Spectroscopy (XRF) for Metallome Analysis of Herbarium Specimens, Plant Methods, 2022, 18 (1), 139. 10.1186/s13007-022-00958-z 36536435 PMC9761992

[40] Solé V. A., Papillon E., Cotte M., Walter P., Susini J., A Multiplatform code for the Analysis of Energy-Dispersive X-Ray Fluorescence Spectra, Spectrochim. Acta Part B, 2007, 62 (1), 63. 10.1016/j.sab.2006.12.002

[41] Schindelin J., Arganda-Carreras I., Frise E., Kaynig V., Longair M., Pietzsch T., Preibisch S., Rueden C., Saalfeld S., Schmid B., Tinevez J. Y., Fiji: an Open-Source Platform for Biological-Image Analysis, Nat. Methods, 2012, 9 (7), 676. 10.1038/nmeth.2019 22743772 PMC3855844

[42] Corzo Remigio A., Nkrumah P. N., Pošćić F., Edraki M., van der Ent A., Comprehensive Insights in Thallium Ecophysiology in the Hyperaccumulator *Biscutella laevigata*, Sci. Total Environ., 2022, 838 (2), 155899. 10.1016/j.scitotenv.2022.155899 35569660

